# From fellows to leaders: lessons learned from the WHO/TDR Clinical Research and Development Fellowship (1999–2021)

**DOI:** 10.5588/pha.26.0010

**Published:** 2026-05-18

**Authors:** M. Vahedi, D.G. Assefa, N. Casamitjana, P. Launois

**Affiliations:** 1 World Health Organization, The Special Programme for Research and Training in Tropical Diseases (WHO/TDR), Geneva, Switzerland;; 2 ISGlobal, University of Barcelona, Barcelona, Spain;; 3 Department of Nursing, College of Health Science and Medicine, Dilla University, Dilla, Ethiopia.

**Keywords:** research leadership development, clinical research, WHO/TDR

## Abstract

**BACKGROUND:**

Strengthening research leadership in low- and middle-income countries (LMICs) is essential for addressing diseases of poverty. Since 1999, World Health Organization (WHO) Special Programme for Research and Training in Tropical Diseases (TDR) has supported this through the Clinical Research and Development Fellowship (CRDF), but evidence on its long-term impact remains limited. We therefore assessed the leadership trajectories of CRDF fellows trained between 1999 and 2021.

**METHODS:**

An online cross-sectional survey of CRDF alumni (1999–2021) used a structured questionnaire capturing career progression, productivity, leadership roles, collaborations, institutional contributions, and challenges.

**RESULTS:**

Of 116 fellows contacted, 88 responded (75.8%). More than half had secured competitive funding (55.7%), with a median grant value of USD 512,320 (interquartile range: 111,629–1,200,000; range: USD 700–70,000,000), including multiple national and international awards. Career advancement was notable: 43.2% served as principal investigators and nearly 80% supervised research staff. Many led developments of infrastructure and operating procedures (63.6%). Re-entry grants strengthened institutional capacity for respondents. Collaborations were sustained nationally (77.3%) and through South–South partnerships (71.6%). Almost all fellows (98.9%) continued applying TDR-acquired leadership skills. One third contributed to policy documents. Persistent barriers included difficulty accessing funding, limited institutional support, scarce national funding, and heavy workloads.

**CONCLUSION:**

CRDF alumni reported substantial engagement in research leadership, scientific productivity, and institutional capacity strengthening in LMICs.

Infectious diseases continue to disproportionately affect populations living in poverty, particularly in low- and middle-income countries (LMICs).^1^ Despite increased global investment in health research and development, substantial inequities persist in who sets research agendas, governs the production of knowledge, and translates evidence into health policy and innovation. In many LMICs, weak institutional environments and limited national research leadership constrain the generation and application of context-responsive evidence.^2,3^ These limitations contribute to continued dependence on external sources for health technologies, including vaccines, pharmaceuticals,^4^ and diagnostics,^5^ and reflect broader gaps in regulatory expertise, research workforce capacity, and governance.^6^ The COVID-19 pandemic renewed attention to local and regional research and development capacity, but infrastructure alone is insufficient.^6,7^ Without capable research leaders able to generate evidence, manage multidisciplinary teams, navigate regulatory systems, and engage policymakers, investments in innovation are unlikely to translate into sustainable and equitable access to health technologies.^6,7^

Recognising the importance of research leadership in LMICs, the WHO Special Programme for Research and Training in Tropical Diseases (TDR) has long supported research capacity strengthening. One important initiative is the Clinical Research and Development Fellowship (CRDF) programme, launched in 1999.^8,9^ The CRDF provides early- and mid-career researchers and clinicians from LMICs with approximately 1 year of placement-based training at international Training Partner Organisations (TPOs), including academic-affiliated research organisations, pharmaceutical companies, and product development partnerships.^8,9^ The programme focuses on clinical research, regulatory science, trial management, and research governance, with fellows expected to return to their home institutions after training.^10^ Although short-term outputs of research training programmes are relatively well documented, evidence on mid- and long-term outcomes remains limited.^9,11^ Existing evaluations often emphasise publications while under-capturing leadership trajectories, institutional contributions, collaboration patterns, and sustainability conditions central to WHO/TDR and Enhancing Support for Strengthening the Effectiveness of National Capacity Efforts (ESSENCE) frameworks.^12^

We therefore aimed to describe the mid- and long-term career trajectories, leadership roles, collaboration patterns, and institutional engagements of WHO/TDR CRDF fellows trained between 1999 and 2021.

## METHODS

We conducted an online cross-sectional survey of alumni of the WHO/TDR CRDF programme. Eligible participants were fellows who completed the programme between 1999 and 2021 and returned to institutions in LMICs. All CRDF fellows listed in the TDR alumni database were invited to participate. Participation was voluntary, and informed consent was obtained electronically at survey initiation. Data were anonymised and accessible only to the study team.

### Survey instrument and data collection

A structured survey was developed in alignment with WHO/TDR priorities and the ESSENCE framework for research capacity strengthening. It captured outcomes at three levels: i) individual research capacity and leadership development; ii) institutional research capacity strengthening; and iii) the broader research ecosystem, including collaboration and network sustainability. The survey included both closed- and open-ended items.

The survey was administered through Google Forms from 28 October to 12 December 2025. Four reminder emails and five WhatsApp reminders were sent during the data collection period. To improve data credibility, respondents were invited to provide ORCID identifiers and Google Scholar profiles, and publication records were cross-checked where available. The questionnaire also underwent expert review and pilot testing before use.

### Data analysis

Quantitative data were analysed using R.^13^ Descriptive statistics were used to summarise participant characteristics, leadership roles, collaboration patterns, and reported institutional contributions. Given the exploratory objective, modest sample size, and wide distribution of fellows across cohorts and regions, the analysis focused on descriptive summaries rather than inferential modelling. Likert-scale responses were treated as ordinal, and qualitative responses were analysed thematically to contextualise quantitative findings. Geographic distribution was classified according to WHO regional groupings.^14^ Findings are interpreted as descriptive programme-associated trajectories rather than causal effects.

### Ethical statement

Participation was voluntary, and respondents were permitted to withdraw at any time. Ethical considerations included informed consent at the start of the survey, where participants were notified of the study’s purpose. To ensure confidentiality, all individual data were restricted to the study team, and data were anonymised.

## RESULTS

Among 128 CRDF fellows identified in the TDR database, two were deceased and 10 could not be contacted because of invalid email addresses. Of the remaining 116 fellows contacted, 88 responded, yielding a response rate of 75.8% (Supplementary Data Figure S1). Respondents completed the fellowship between 1999 and 2021; at the time of the 2025 survey, the median time since fellowship completion was 7 years (interquartile range [IQR]: 4–11; range: 2–25). Among respondents, 28.4% (25/88) were women and 71.6% (63/88) were men. The median age at programme entry was 36.0 years (IQR: 34.0–39.0; range: 23.0–50.0), and the median fellowship duration was 1.00 year (IQR: 0.99–1.08; range: 0.09–2.01). Most respondents were from the WHO African Region (76.1%, 67/88), followed by the Region of the Americas (7.9%, 7/88), with smaller proportions from South-East Asia, and the Eastern Mediterranean (Supplementary Data Figure S2).

After the fellowship, 52.2% (46/88) attained a PhD/DPhil, 31.8% (28/88) held a master’s or diploma, and 6.8% (6/88) held an MD. Most alumni remained in their country of citizenship (70.5%, 62/88), while 29.5% (26/88) resided elsewhere. Employment was mainly within universities or academic institutions (34.1%, 30/88) and national research institutes (25.0%, 22/88), followed by NGOs or non-profit organisations (13.6%, 12/88), ministries of health (9.1%, 8/88), and international research institutes (6.8%, 6/88). At the time of the survey, 21.6% (19/88) were living in high-income countries, mainly for doctoral training or research fellowships. Nearly half were mid-career researchers (47.7%, 42/88), while 30.7% (27/88) occupied established or senior leadership positions (Table 1).

**TABLE 1.** Demographic and professional characteristics of fellowship recipients (N = 88).

Characteristics	N = 88
Year of fellowship start	
2000–2010	9 (10.2%)
2010–2014	19 (21.6%)
2015–2018	31 (35.2%)
2019–2023	29 (32.9%)
Current citizenship by subregion	
Eastern Africa	30 (34.1%)
Eastern Asia	1 (1.1%)
Middle Africa	7 (7.9%)
Northern Africa	2 (2.3%)
South America	7 (7.9%)
South-Eastern Asia	1 (1.1%)
Southern Africa	1 (1.1%)
Southern Asia	7 (7.9%)
Western Africa	30 (34.1%)
Western Europe	2 (2.3%)
Highest academic degree before fellowship	
Master’s	26 (29.5%)
MD	34 (38.6%)
Medical specialist	1 (1.1%)
PhD	27 (30.7%)
Highest academic degree after fellowship	
Fellow of the higher education academy	1 (1.1%)
Master’s/diploma	28 (31.8%)
MD	6 (6.8%)
Medical specialist	1 (1.1%)
None	4 (4.5%)
PhD/DPhil	46 (52.2%)
Postdoctoral	1 (1.1%)
Home institution type before fellowship	
Hospital/clinical setting	8 (9.1%)
Ministry of Health/government agency	8 (9.1%)
National research institute	24 (27.2%)
NGO/non-profit	10 (11.4%)
Another research institute	1 (1.1%)
Research institution	1 (1.1%)
University/academic institution	36 (41.0%)
Home institution after fellowship	
Hospital/clinical setting	3 (3.4%)
International research institute	6 (6.8%)
Ministry of Health/government agency	8 (9.1%)
National research institute	22 (25.0%)
NGO/non-profit	12 (13.6%)
Pharmaceutical companies	5 (5.7%)
Private research centre	–
Product development companies	1 (1.1%)
Other (not specific)	–
Unemployed	1 (1.1%)
University/academic institution	30 (34.1%)
Current residence	
Eastern Africa	30 (34.1%)
Eastern Asia	1 (1.1%)
Middle Africa	7 (8.0%)
Northern Africa	2 (2.3%)
South America	7 (8.0%)
South-Eastern Asia	1 (1.1%)
Southern Africa	1 (1.1%)
Southern Asia	7 (8.0%)
Western Africa	30 (34.1%)
Western Europe	2 (2.3%)

### Post-fellowship scientific productivity and leadership

More than half of respondents (55.7%, 49/88) reported securing at least one competitive research grant after completing the fellowship. Among grant recipients, 34.7% (17/49) obtained at least one international grant and 26.5% (13/49) at least one national grant. The median total amount secured post-fellowship was USD 512,320 (IQR: 111,629–1,200,000; range: 700–70,000,000) – see Table 2. In addition, 28.4% (25/88) reported securing research funding through fellowship-enabled networks; among them, the median amount obtained was USD 300,000 (IQR: 50,000–850,000; range: 4,500–100,000,000) – see Table 3.

**TABLE 2.** Post-fellowship outcomes: clinical research capacity, competitive funding, and policy engagement (N = 88).

Characteristics	N = 88
Extent to which the re-entry grant strengthened clinical research capacity	
Not at all enabled	16 (18.2%)
Slightly enabled	–
Moderately enabled	18 (20.5%)
Substantially enabled	35 (39.8%)
Fully enabled	19 (21.6%)
Initiatives implemented at home institution post-fellowship^A^	
Clinical trial activities	3 (3.4%)
Policy and government advisory	23 (26.1%)
SOPs	61 (69.3%)
Training programmes	67 (76.1%)
Other (not specific)	18 (20.5%)
Competitive research grants obtained post-fellowship	
Yes	49 (55.7%)
No	39 (44.3%)
Number of national grants obtained post-fellowship	N = 49
1	13 (26.5%)
2	9 (18.4%)
3	4 (8.2%)
4	1 (2.0%)
5+	4 (8.2%)
Missing	8 (16.3%)
Number of international grants obtained post-fellowship	N = 49
1	17 (34.7%)
2	7 (14.3%)
3	8 (16.3%)
4	2 (4.1%)
5+	9 (18.4%)
Missing	6 (12.2%)
Amount of grant in USD median (IQR1–IQR3) (Min–Max)	512,320 (111,629–1,200,000) (700–70,000,000)
Type of grant^A^	N = 49
Capacity building grant (for training/infrastructure)	28 (57.1%)
Research grant (for a specific study)	42 (85.7%)
Research grant; capacity-building grant (combined)	18 (36.7%)
Have you published or produced a policy brief or policy document based on your research findings?	N = 88
Yes	29 (33.0%)
No	59 (67.0%)
How many policies brief or policy document published or produced	N = 29
1	7 (24.1%)
2	14 (48.3%)
4	1 (3.4%)
6	1 (3.4%)
15	1 (3.4%)
Multiple (number not specified)	5 (17.2%)
How many implemented	N = 29
1	7 (24.1%)
2	10 (34.5%)
3	1 (3.4%)
Multiple (number not specified)	11 (37.9%)

IQR = interquartile range.

A Participants could select multiple answers; percentages are calculated within each fellowship type.

**TABLE 3.** Collaboration with the training partner organisation after fellowship.

Collaboration with TPO after fellowship	N = 88
Collaboration with individual members of the TPO (e.g., supervisors, mentors, and lab teams)	11 (12.5%)
Collaboration with partners or networks you were introduced to through the TPO during the fellowship	7 (8.0%)
Direct collaboration with the TPO	4 (4.5%)
Direct collaboration with the TPO; collaboration with individual members of the TPO (e.g., supervisors, mentors, and lab teams)	9 (10.2%)
Direct collaboration with the TPO; collaboration with individual members of the TPO (e.g., supervisors, mentors, and lab teams); collaboration with partners or networks you were introduced to through the TPO during the fellowship	14 (16.9%)
Direct collaboration with the TPO; collaboration with partners or networks you were introduced to through the TPO during the fellowship	7 (8.0%)
Direct collaboration with the TPO; The African Field Epidemiology Network (AFENET) and the Institute Pasteur de Dakar of Senegal	1 (1.1%)
Other collaboration; direct collaboration with the TPO; Was later hired as a Clinical Science Lead in my training institution in Vaccines R&D.	1 (1.1%)
Other collaboration; EDCTP Institutional Capacity Building grant and the malaria development consortium grant	1 (1.1%)
No collaboration	29 (33.5%)
Nature of collaboration with TPO	N = 59
Co-authored publications	15 (25.4%)
Joint research projects (including clinical trials)	14 (23.7%)
Grant proposal development/submissions	12 (20.3%)
Training activities/capacity building	11 (18.6%)
Mentorship (formal or informal)	9 (15.3%)
Consultancy/expert advisory roles	6 (10.2%)
Employment or continued engagement with TPO	5 (8.5%)
Networking/participation in panels, symposia, and programmes	4 (6.8%)
Competitive funding obtained through fellowship networks	N = 88
Yes	27 (30.7%)
No	61 (72.3%)
Estimate the number of grants/contracts secured through this network	N = 27
1	12 (44.4%)
2	7 (25.9%)
3	5 (18.5%)
4	2 (7.4%)
5	1 (3.7%)
No response	–
Estimate the number of grants/contracts secured through this network in USD	N = 25
Median (IQR1–IQR3) (Min–Max)	300,000 (50,000–850,000) (4,500–100,000,000)
Type of collaboration (North–North/North–South)	N = 88
Never	8 (9.1%)
Rarely	12 (13.6%)
Sometimes	31 (35.2%)
Very often	37 (42.0%)
Type of collaboration (South–South)	
Never	8 (9.1%)
Rarely	17 (19.3%)
Sometimes	33 (37.5%)
Very often	30 (34.1%)
Type of collaboration (national)	
Never	4 (4.5%)
Rarely	16 (18.2%)
Sometimes	32 (36.4%)
Very often	36 (40.9%)
Evolution in authorship	N = 88
Evolution in first authorship positions after the fellowship compared to before	
No change	13 (14.8%)
Minimal improvement	5 (5.7%)
Moderate improvement	12 (13.6%)
Substantial improvement	22 (22.0%)
Significant improvement	36 (40.9%)
Evolution in last/senior authorship positions after the fellowship compared to before	
No change	25 (28.4%)
Minimal improvement	7 (8.0%)
Moderate improvement	13 (14.8%)
Substantial improvement	17 (19.3%)
Significant improvement	26 (29.5%)
Evolution in corresponding authorship positions after the fellowship compared to before	
No change	13 (14.8%)
Minimal improvement	4 (4.5%)
Moderate improvement	13 (14.8%)
Substantial improvement	27 (30.7%)
Significant improvement	31 (35.2%)

EDCTP = European & Developing Countries Clinical Trials Partnership; TPO = Training Partner Organisation; IQR = interquartile range.

Publication records were verified through ORCID and Google Scholar where available. Among the 88 respondents, 76 (86.4%) had verifiable publication records and had collectively contributed 1,821 peer-reviewed publications. Of these fellows, 17 (22.4%) were women and 59 (77.6%) were men. Male fellows contributed 1,494 publications (82.0%), while female fellows contributed 327 (18.0%). Most publications were attributed to fellows from the WHO African Region (79.4%), followed by the Americas (12.5%), South-East Asia (5.8%), and the Western Pacific (2.3%). The median number of publications per fellow was 16 (IQR: 7–30; range: 1–135). Authorship trajectories also suggested professional progression. Improvements in first authorship were reported by 62.9% (58/88) of alumni, while 48.8% (43/88) reported substantial or significant improvement in senior or last authorship roles (Table 3).

At the time of the survey, 43.2% (38/88) were serving as principal investigators or lead researchers. Alumni also reported holding positions as research group or team leaders (31.8%, 28/88), academic faculty (30.7%, 27/88), heads of department (18.2%, 16/88), leaders of research units (15.9%, 14/88), and project managers (20.4%, 18/88) – see Table 4. A large majority (79.5%, 70/88) reported direct management or supervision of research staff or students, often in small- to medium-sized teams.

**TABLE 4.** Current professional roles and supervisory responsibilities of fellows (N = 88).

Current position of the fellows^A^	N = 88
Academic faculty (lecturer/assistant professor/professor)	27 (30.7%)
Associate Director General	1 (1.1%)
Associate Director of NGO	1 (1.1%)
Biostatistician	1 (1.1%)
Chairperson of a research ethics committee	1 (1.1%)
Clinical Data Manager	1 (1.1%)
Clinical Development Physician (director level in the pharmaceutical industry)	1 (1.1%)
Clinical Research Scientist/Clinical Science Lead/Safety Physician	1 (1.1%)
Clinical Trial Coordinator	11 (12.5%)
Clinical Trial Manager	8 (9.1%)
Clinical trial data unit team lead	1 (1.1%)
Disease Surveillance Information System Coordinator	1 (1.1%)
Expert and Lead	1 (1.1%)
Head of Department (university, research institute, or hospital)	16 (18.2%)
Head of Research Unit	14 (15.9%)
Head of clinical department	1 (1.1%)
Lead for a clinical research	1 (1.1%)
Market Developer in clinical research	1 (1.1%)
Policy Advisor/Maker/Implementer	10 (11.4%)
Postdoctoral Researcher	1 (1.1%)
Principal Investigator/Lead Researcher	38 (43.2%)
Programme Coordinator of the Epidemiology and Disease Control Programme, heading the PHEOC and also the Programme Director for The Gambia FETP	1 (1.1%)
Project Manager (research or public health project)	18 (20.4%)
Research Director	10 (11.4%)
Research Group Leader/Team Leader	28 (31.8%)
Responsible for clinical operations	1 (1.1%)
Safety lead for a global vaccine programme	1 (1.1%)
Strategy and business development in clinical research	1 (1.1%)
Technical Officer in Foresight Leadership and Ethics in Science Unit	1 (1.1%)
Therapeutic area leads drug safety (Exec Director) in a pharmaceutical company	1 (1.1%)
Direct management of other research staff/students	
Yes	70 (79.5%)
No	18 (20.5%)
Number of staff/students managed by the fellow	N = 70
1	1 (1.4%)
2–5	23 (32.9%)
6–10	17 (24.3%)
11–20	12 (17.1%)
21–50	4 (5.7%)
51	4 (5.7%)
No response	18 (25.7%)
Level of staff/students managed by the fellow	N = 70
Junior/early career	1 (1.1%)
Mid-level/MSc	4 (5.7%)
PhD/MD level	4 (5.7%)
Unspecified level	25 (35.7%)
No response	36 (51.4%)
Direct supervision of other research staff/students	
Yes	70 (79.5%)
No	18 (20.5%)
Number of staff/students supervised by the fellow	N = 70
1–2	7 (10.0%)
3–5	22 (31.4%)
6–10	20 (28.6%)
11–20	10 (14.3%)
20+	2 (2.9%)
No response	9 (12.9%)
Level of staff/students supervised by the fellow	N = 70
Junior/early career	22 (31.4%)
Mid-level/MSc	9 (12.9%)
PhD/MD level	5 (7.1%)
Unspecified level	5 (7.1%)
No response	29 (41.4%)

A Participants could select multiple roles; percentages are calculated within each fellowship type.

PHEOC = Public Health Emergency Operations Centre; FETP = Field Epidemiology Training Programme.

### Leadership confidence, collaboration, and institutional contributions

Self-reported confidence in leadership and research governance was high. Around two thirds reported being very or extremely confident in engaging policymakers and regulators (65.9%, 58/88), managing budgets and financial governance (65.9%, 58/88), leading multidisciplinary clinical research teams (69.3%, 61/88), and developing clinical trial protocols (73.8%, 65/88) (Supplementary Data Table S1). Similarly, 84.1% (74/88) reported high confidence in team management and 81.8% (72/88) in leading meetings effectively (Supplementary Data Table S2).

Collaboration after the fellowship remained common. Overall, 66.5% (59/88) reported ongoing collaboration with their TPO, often through co-authored publications, joint research projects, and grant development (Table 3). Frequent collaboration was also reported at national level (40.9%, 36/88), through South–South partnerships (34.1%, 30/88) and North–South collaborations (42.0%, 37/88). Mentorship was rated highly during the fellowship, and post-fellowship mentorship remained important, with 44.3% (39/88) describing it as essential and 38.6% (34/88) as very useful (Supplementary Data Table S3).

Nearly all alumni (98.9%, 87/88) reported actively using fellowship-acquired skills in their current positions. Confidence was particularly high in strategic planning for clinical research programmes (75.0%, 66/88), mentoring junior researchers (78.4%, 69/88), and supervising junior staff (84.1%, 74/88) (Supplementary Data Table S1). More than three in five fellows (61.4%, 54/88) reported that re-entry grants substantially or fully enabled strengthening of clinical research capacity at their home institutions. Frequently reported institutional contributions included establishing training programmes (76.1%, 67/88) and developing standard operating procedures (69.3%, 61/88) – see Table 2. In addition, one third (33.0%, 29/88) reported producing at least one policy brief or policy document based on their research.

### Barriers and sustainability challenges

Respondents also identified important barriers. Before placement, 26.1% (23/88) reported challenges such as delays in visas and flight arrangements. During placement, 33.0% (29/88) reported logistical barriers including housing and banking. Despite this, 86.4% (76/88) perceived access to resources at TPOs as mostly or fully equitable compared with peers at their home institutions (Supplementary Data Table S4). After the fellowship, the most common re-entry challenges were lack of research funding (39.8%, 35/88), limited institutional support or buy-in (20.5%, 18/88), and heavy service or teaching workloads (11.4%, 10/88) (Figure). Respondents also identified unmet needs in grant acquisition, budgeting, financial planning, and team leadership. More broadly, challenges to grant acquisition were described as systemic, including limited knowledge of funders (70.5%, 62/88), research capacity and environmental constraints (51.3%, 45/88), inadequate institutional grant support (50.0%, 44/88), and limited national funding opportunities (43.2%, 38/88) – see Supplementary Data Tables S5–S7. The TDR fellowship itself was rated as extremely important to professional success by 59.1% (52/88), followed by family support, mentors, supervisors, and employer or home institution support (Supplementary Data Table S3).

**FIGURE.**
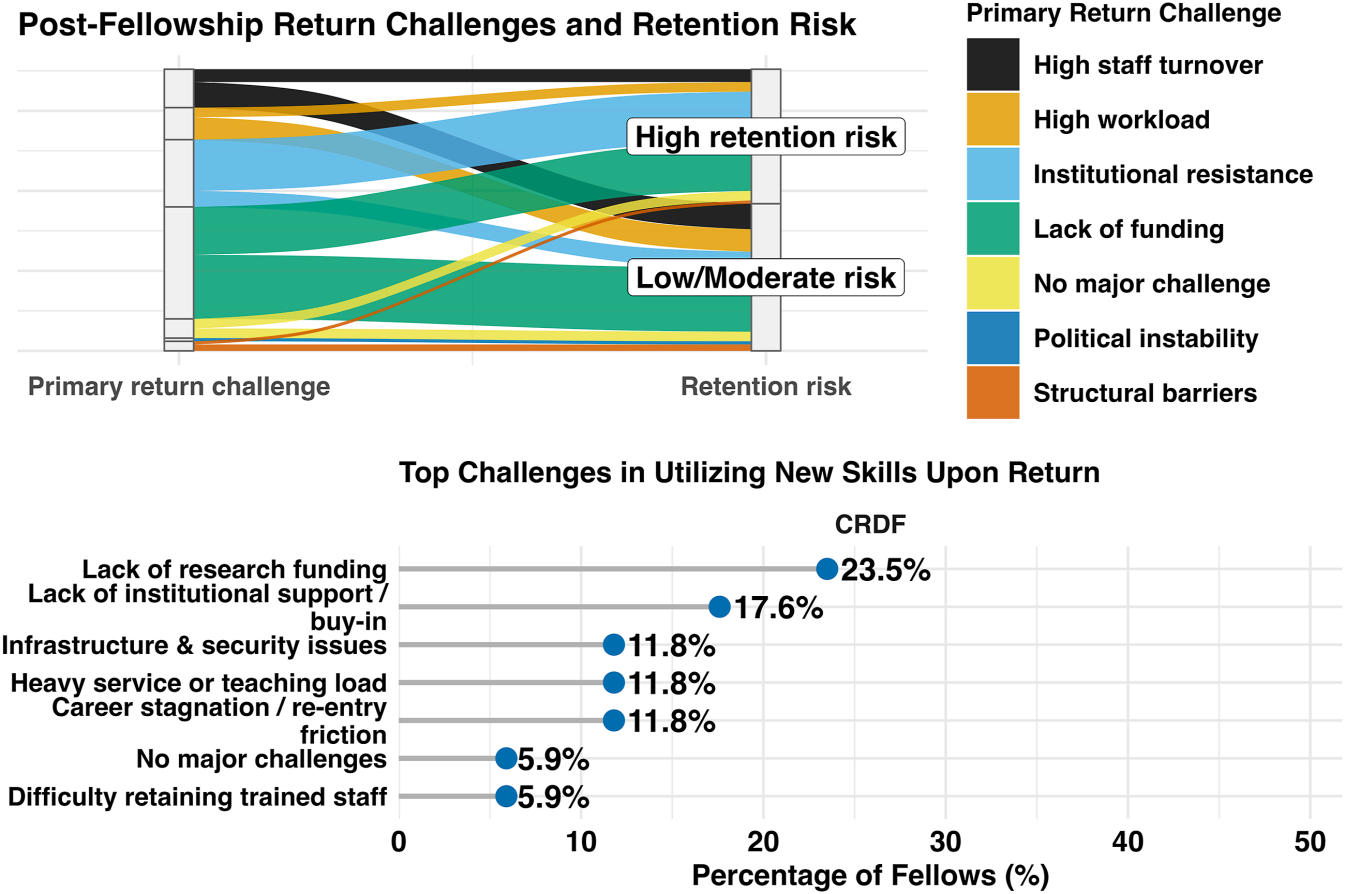
Post-fellowship return challenges and retention risk and challenges in utilising new skill upon return.

## DISCUSSION

This study provides descriptive evidence on the reported mid- and long-term professional trajectories of WHO/TDR CRDF alumni across research leadership, scientific productivity, collaboration, and institutional engagement. Overall, the findings are consistent with broader research capacity-strengthening literature showing that fellowship programmes can support skill development, scientific productivity, and career progression, especially when training is linked to mentorship, networks, and continued opportunities after programme completion.^8,9^ In the present study, many alumni reported progression into leadership roles, supervision of junior researchers, acquisition of grants, and sustained collaboration with training partners. The high proportion of respondents reporting leadership roles may partly reflect the design of the CRDF programme, which targets researchers and clinicians already positioned to contribute to research implementation and institutional leadership in their home settings.^8,9^ The programme’s practical placement-based model may also explain why many alumni reported confidence in protocol development, team management, research governance, and stakeholder engagement.^10^ This aligns with literature suggesting that programmes combining technical training, embedded mentorship, and real-world exposure are more likely to produce durable professional gains than short-term classroom-based training alone.^15^

Continued collaboration with TPOs and broader research networks was another important finding. The persistence of co-authorship, joint projects, and grant development suggests that the value of the fellowship extends beyond individual training to network formation and sustained professional connectivity. This is particularly relevant in LMIC settings, where constrained infrastructure and limited access to funding may make international and regional collaboration essential for maintaining scientific productivity and leadership development.^9,11^ The prominence of national and South–South collaboration in this study further supports the view that regional networks can complement North–South partnerships by strengthening local ownership, contextual relevance, and more equitable leadership.^16^

At the same time, the study identified substantial institutional and systemic constraints after fellowship completion. Lack of research funding, weak institutional support, competing workloads, and limited grant-management environments were common challenges. These findings are consistent with WHO/TDR and ESSENCE guidance,^12^ which emphasise that individual-level training alone is insufficient when institutional absorptive capacity is weak.^17,18^ In this context, the gains associated with fellowships may depend not only on the quality of training but also on whether home institutions could absorb, support, and sustain newly developed expertise.^4,19,20^

Our findings therefore suggest that the contribution of the CRDF programme is best understood as part of a broader capacity-strengthening pathway linking individual development with institutional opportunity and research ecosystem support. Many of the reported competencies, including trial governance, mentorship, stakeholder engagement, and team leadership, are also transferable beyond infectious disease research. However, because the study is cross-sectional and relies largely on self-reported data, these findings should be interpreted as descriptive programme-associated patterns rather than causal effects.

A key strength of this evaluation is its focus on mid- and long-term trajectories rather than immediate post-training outputs. It also used a broad monitoring and evaluation framework spanning leadership, productivity, collaboration, and institutional engagement. However, several limitations should be acknowledged. Findings rely primarily on self-reported data and are therefore subject to recall and social desirability bias. The cross-sectional design and absence of a counterfactual group preclude causal inference. Publication outputs could be verified where ORCID or Google Scholar records were available, but other outcomes were not independently validated. In addition, the predominance of respondents from the WHO African Region reflects the programme’s historical orientation and limits generalisability across all LMIC regions. The descriptive analytical approach also did not assess statistical associations between demographic characteristics and outcomes.

This study contributes to development practice by providing empirically grounded insights into how fellowship-based capacity strengthening interacts with institutional environments in resource constrained settings. It highlights human resource leadership, institutional absorptive capacity, and South–South collaboration as critical enablers of sustainable, locally led research systems. These insights are directly relevant to development agencies, funders, and national governments designing long-term research capacity-strengthening interventions.

## CONCLUSION

This study provides descriptive insights into the reported mid- and long-term professional trajectories of WHO/TDR CRDF alumni, particularly within African LMIC contexts. Findings suggest that many alumni progressed into research leadership roles, sustained scientific productivity, contributed to institutional research capacity, and remained engaged in collaborative research networks. At the same time, persistent structural constraints, including limited funding and institutional support, may affect the sustainability of these gains. These findings highlight the potential value of pairing individual fellowships with re-entry support, protected research time, stronger research management systems, and South–South collaboration to support more sustainable and locally led research ecosystems.

## Supplementary Material
