# Supplementary material for: From fellows to leaders: lessons learned from the WHO/TDR Clinical Research and Development Fellowship (1999–2021)
Source: Public Health Action. 2026 May 18;16(2):86–93. doi: 10.5588/pha.26.0010 (PMC13182883; doi:10.5588/pha.26.0010)
Supplement: Supplementary file 1 [file pha26-0010_supplementarydata1.pdf]

From Fellows to Leaders: Lessons learned from the WHO/TDR Clinical Research and Development Fellowship (1999–2021)

Mahnaz Vahedi[1,4]*, Dawit Getachew Assefa[2], Nuria Casamitjana[3], Pascal Launois[1]

[1]World Health Organization, The Special Programme for Research and Training in Tropical Diseases (WHO/TDR), Geneva, Switzerland

[2]Department of Nursing, College of Health Science and Medicine, Dilla University, Dilla, Ethiopia

[3]IS Global, University of Barcelona, Barcelona, Spain

Corresponding author*: Dr Mahnaz Vahedi , WHO/TDR and University of Barcelona, Email: vahedim@who.int; mvahedva7@alumnes.ub.edu

Abstract

Background: Strengthening research leadership in low- and middle-income countries (LMICs) is essential for addressing diseases of poverty. Since 1999, WHO/TDR has supported this through the Clinical Research and Development Fellowship (CRDF). However, evidence on its long-term impact remains limited. This study assessed leadership trajectories of CRDF fellows trained between 1999 and 2021.

Methods: An online cross-sectional survey of CRDF alumni (1999–2021) used a structured questionnaire capturing career progression, productivity, leadership roles, collaborations, institutional contributions, and challenges.

Results: Of 116 fellows contacted, 88 responded (75.8%). More than half secured competitive funding (55.7%), with a median grant value of USD 512,320 (IQR: 111,629–1,200,000; range: USD 700–70,000,000), including multiple national and international awards. Career advancement was notable: 43.2% served as principal investigators and nearly 80% supervised research staff. Many led developments of infrastructure and operating procedures (63.6%). Re-entry grants strengthened institutional capacity for respondents. Collaborations were sustained nationally (77.3%) and through South–South partnerships (71.6%). Almost all fellows (98.9%) continued applying TDR-acquired leadership skills. One-third contributed to policy documents. Persistent barriers included difficulty accessing funding, limited institutional support, scarce national funding, and heavy workloads.

Conclusions: CRDF alumni reported substantial engagement in research leadership, scientific productivity, and institutional capacity strengthening in LMIC settings.

Key words: Research Leadership Development; Clinical Research; WHO/TDR

Introduction

Infectious diseases continue to disproportionately affect populations living in poverty, particularly in low- and middle-income countries (LMICs) (1). Despite increased global investment in health research and development, substantial inequities persist in who sets research agendas, governs the production of knowledge, and translates evidence into health policy and innovation. In many LMICs, weak institutional environments and limited national research leadership constrain the generation and application of context-responsive evidence (2,3).These limitations contribute to continued dependence on external sources for health technologies, including vaccines, pharmaceuticals (4), and diagnostics (5), and reflect broader gaps in regulatory expertise, research workforce capacity, and governance (6).

The COVID-19 pandemic renewed attention to local and regional research and development capacity, but infrastructure alone is insufficient (6,7). Without capable research leaders able to generate evidence, manage multidisciplinary teams, navigate regulatory systems, and engage policymakers, investments in innovation are unlikely to translate into sustainable and equitable access to health technologies (6,7).

Recognising the importance of research leadership in LMICs, the World Health Organization (WHO) Special Programme for Research and Training in Tropical Diseases (TDR) has long supported research capacity strengthening. One important initiative is the Clinical Research and Development Fellowship (CRDF) Programme, launched in 1999 (8,9). The CRDF provides early- and mid-career researchers and clinicians from LMICs with approximately one year of placement-based training at international Training Partner Organisations (TPOs), including academic-affiliated research organisations, pharmaceutical companies, and product development

partnerships (PDP) (8,9).The programme focuses on clinical research, regulatory science, trial management, and research governance, with fellows expected to return to their home institutions after training (10).

Although short-term outputs of research training programmes are relatively well documented, evidence on mid- and long-term outcomes remains limited (9,11). Existing evaluations often emphasise publications while under-capturing leadership trajectories, institutional contributions, collaboration patterns, and sustainability conditions central to WHO/TDR and Enhancing Support for strengthening the effectiveness of national capacity efforts (ESSENCE) frameworks (12). This study therefore aimed to describe the mid- and long-term career trajectories, leadership roles, collaboration patterns, and institutional engagements of WHO/TDR CRDF fellows trained between 1999 and 2021.

Methodology

Study design and population

We conducted an online cross-sectional survey of alumni of the WHO/TDR CRDF Programme. Eligible participants were fellows who completed the programme between 1999 and 2021 and returned to institutions in LMICs. All CRDF fellows listed in the TDR alumni database were invited to participate. Participation was voluntary, and informed consent was obtained electronically at survey initiation. Data were anonymised and accessible only to the study team.

Survey instrument and data collection

A structured survey was developed in alignment with WHO/TDR priorities and the ESSENCE framework for research capacity strengthening. It captured outcomes at three levels: (i) individual research capacity and leadership development; (ii) institutional research capacity strengthening; and (iii) the broader research ecosystem, including collaboration and network sustainability. The survey included both closed- and open-ended items.

The survey was administered through Google Forms from 28 October to 12 December 2025. Four reminder emails and five WhatsApp reminders were sent during the data collection period. To improve data credibility, respondents were invited to provide ORCID identifiers and Google Scholar profiles, and publication records were cross-checked where available. The questionnaire also underwent expert review and pilot testing before use.

Data analysis

Quantitative data were analysed using R (13). Descriptive statistics were used to summarise participant characteristics, leadership roles, collaboration patterns, and reported institutional contributions. Given the exploratory objective, modest sample size, and wide distribution of fellows across cohorts and regions, the analysis focused on descriptive summaries rather than inferential modelling. Likert-scale responses were treated as ordinal, and qualitative responses were analysed thematically to contextualise quantitative findings. Geographic distribution was classified according to WHO regional groupings (14). Findings are interpreted as descriptive programme-associated trajectories rather than causal effects.

Results

Cohort profile and fellowship exposure

Among 128 CRDF fellows identified in the TDR database, two were deceased and 10 could not be contacted because of invalid email addresses. Of the remaining 116 fellows contacted, 88 responded, yielding a response rate of 75.8% (**Supplementary Figure 1**). Respondents completed the fellowship between 1999 and 2021; at the time of the 2025 survey, the median time since fellowship completion was 7 years (IQR: 4–11; range: 2–25 years).

Among respondents, 28.4% (25/88) were women and 71.6% (63/88) were men. The median age at programme entry was 36.0 years (IQR: 34.0–39.0; range: 23.0–50.0), and the median fellowship duration was 1.00 year (IQR: 0.99–1.08; range: 0.09–2.01). Most respondents were from the WHO African Region (76.1%, 67/88), followed by the Region of the Americas (7.9%, 7/88), with smaller proportions from South-East Asia and the Eastern Mediterranean (**Supplementary Figure 2**).

After the fellowship, 52.2% (46/88) attained a PhD/DPhil, 31.8% (28/88) held a Master's or Diploma, and 6.8% (6/88) held an MD. Most alumni remained in their country of citizenship (70.5%, 62/88), while 29.5% (26/88) resided elsewhere. Employment was mainly within universities or academic institutions (34.1%, 30/88) and national research institutes (25.0%, 22/88), followed by NGOs or non-profit organisations (13.6%, 12/88), ministries of health (9.1%, 8/88), and international research institutes (6.8%, 6/88). At the time of the survey, 21.6% (19/88) were living in high-income countries, mainly for doctoral training or research fellowships. Nearly half were mid-career researchers (47.7%, 42/88), while 30.7% (27/88) occupied established or senior leadership positions (**Table 1**).

Post-fellowship scientific productivity and leadership

More than half of respondents (55.7%, 49/88) reported securing at least one competitive research grant after completing the fellowship. Among grant recipients, 34.7% (17/49) obtained at least one international grant and 26.5% (13/49) at least one national grant. The median total amount secured post-fellowship was USD 512,320 (IQR: 111,629–1,200,000; range: 700–70,000,000) (**Table 2**). In addition, 28.4% (25/88) reported securing research funding through fellowship-enabled networks; among them, the median amount obtained was USD 300,000 (IQR: 50,000–850,000; range: 4,500–100,000,000) (**Table 3**).

Publication records were verified through ORCID and Google Scholar where available. Among the 88 respondents, 76 (86.4%) had verifiable publication records and had collectively contributed 1,821 peer-reviewed publications. Of these fellows, 17 (22.4%) were women and 59 (77.6%) were men. Male fellows contributed 1,494 publications (82.0%), while female fellows contributed 327 (18.0%). Most publications were attributed to fellows from the WHO African Region (79.4%),

followed by the Americas (12.5%), South-East Asia (5.8%), and the Western Pacific (2.3%). The median number of publications per fellow was 16 (IQR: 7–30; range: 1–135).

Authorship trajectories also suggested professional progression. Improvements in first authorship were reported by 62.9% (58/88) of alumni, while 48.8% (43/88) reported substantial or significant improvement in senior or last authorship roles (**Table 3**).

At the time of the survey, 43.2% (38/88) were serving as principal investigators or lead researchers. Alumni also reported holding positions as research group or team leaders (31.8%, 28/88), academic faculty (30.7%, 27/88), heads of department (18.2%, 16/88), leaders of research units (15.9%, 14/88), and project managers (20.4%, 18/88) (**Table 4**). A large majority (79.5%, 70/88) reported direct management or supervision of research staff or students, often in small- to medium-sized teams.

Leadership confidence, collaboration, and institutional contributions

Self-reported confidence in leadership and research governance was high. Around two-thirds reported being very or extremely confident in engaging policymakers and regulators (65.9%, 58/88), managing budgets and financial governance (65.9%, 58/88), leading multidisciplinary clinical research teams (69.3%, 61/88), and developing clinical trial protocols (73.8%, 65/88) (**Supplementary Table 1**). Similarly, 84.1% (74/88) reported high confidence in team management and 81.8% (72/88) in leading meetings effectively (**Supplementary Table 2**).

Collaboration after the fellowship remained common. Overall, 66.5% (59/88) reported ongoing collaboration with their TPO, often through co-authored publications, joint research projects, and grant development (**Table 3**). Frequent collaboration was also reported at national level (40.9%,

36/88), through South–South partnerships (34.1%, 30/88), and North–South collaborations (42.0%, 37/88). Mentorship was rated highly during the fellowship, and post-fellowship mentorship remained important, with 44.3% (39/88) describing it as essential and 38.6% (34/88) as very useful (**Supplementary Table 3**).

Nearly all alumni (98.9%, 87/88) reported actively using fellowship-acquired skills in their current positions. Confidence was particularly high in strategic planning for clinical research programmes (75.0%, 66/88), mentoring junior researchers (78.4%, 69/88), and supervising junior staff (84.1%, 74/88) (**Supplementary Table 1**). More than three in five fellows (61.4%, 54/88) reported that re-entry grants substantially or fully enabled strengthening of clinical research capacity at their home institutions. Frequently reported institutional contributions included establishing training programmes (76.1%, 67/88) and developing standard operating procedures (69.3%, 61/88) (**Table 2**). In addition, one-third (33.0%, 29/88) reported producing at least one policy brief or policy document based on their research.

Barriers and sustainability challenges

Respondents also identified important barriers. Before placement, 26.1% (23/88) reported challenges such as delays in visas and flight arrangements. During placement, 33.0% (29/88) reported logistical barriers including housing and banking. Despite this, 86.4% (76/88) perceived access to resources at TPOs as mostly or fully equitable compared with peers at their home institutions (**Supplementary Table 4**).

After the fellowship, the most common re-entry challenges were lack of research funding (39.8%, 35/88), limited institutional support or buy-in (20.5%, 18/88), and heavy service or teaching workloads (11.4%, 10/88). Respondents also identified unmet needs in grant acquisition, budgeting, financial planning, and team leadership. More broadly, challenges to grant acquisition were described as systemic, including limited knowledge of funders (70.5%, 62/88), research capacity and environmental constraints (51.3%, 45/88), inadequate institutional grant support (50.0%, 44/88), and limited national funding opportunities (43.2%, 38/88) (**Supplementary Tables 5–7**). The TDR fellowship itself was rated as extremely important to professional success by 59.1% (52/88), followed by family support, mentors, supervisors, and employer or home institution support (**Supplementary Table 3**).

Discussion

This study provides descriptive evidence on the reported mid- and long-term professional trajectories of WHO/TDR CRDF alumni across research leadership, scientific productivity, collaboration, and institutional engagement. Overall, the findings are consistent with broader research capacity-strengthening literature showing that fellowship programmes can support skill development, scientific productivity, and career progression, especially when training is linked to mentorship, networks, and continued opportunities after programme completion (8,9). In the present study, many alumni reported progression into leadership roles, supervision of junior researchers, acquisition of grants, and sustained collaboration with training partners.

The high proportion of respondents reporting leadership roles may partly reflect the design of the CRDF programme, which targets researchers and clinicians already positioned to contribute to research implementation and institutional leadership in their home settings (8,9). The programme's practical placement-based model may also explain why many alumni reported confidence in protocol development, team management, research governance, and stakeholder engagement (10). This aligns with literature suggesting that programmes combining technical training, embedded mentorship, and real-world exposure are more likely to produce durable professional gains than short-term classroom-based training alone (15).

Continued collaboration with TPOs and broader research networks was another important finding. The persistence of co-authorship, joint projects, and grant development suggests that the value of the fellowship extends beyond individual training to network formation and sustained professional connectivity. This is particularly relevant in LMIC settings, where constrained infrastructure and limited access to funding may make international and regional collaboration essential for

maintaining scientific productivity and leadership development (9,11). The prominence of national and South–South collaboration in this study further supports the view that regional networks can complement North–South partnerships by strengthening local ownership, contextual relevance, and more equitable leadership (20).

At the same time, the study identified substantial institutional and systemic constraints after fellowship completion. Lack of research funding, weak institutional support, competing workloads, and limited grant-management environments were common challenges. These findings are consistent with WHO/TDR and ESSENCE guidance (12), which emphasise that individual-level training alone is insufficient when institutional absorptive capacity is weak (16,17). In this context, the gains associated with fellowships may depend not only on the quality of training but also on whether home institutions could absorb, support, and sustain newly developed expertise (4,18,19).

The findings therefore suggest that the contribution of the CRDF programme is best understood as part of a broader capacity-strengthening pathway linking individual development with institutional opportunity and research ecosystem support. Many of the reported competencies, including trial governance, mentorship, stakeholder engagement, and team leadership, are also transferable beyond infectious disease research. However, because the study is cross-sectional and relies largely on self-reported data, these findings should be interpreted as descriptive programme-associated patterns rather than causal effects.

Strengths and limitations

A key strength of this evaluation is its focus on mid- and long-term trajectories rather than immediate post-training outputs. It also used a broad monitoring and evaluation framework spanning leadership, productivity, collaboration, and institutional engagement. However, several limitations should be acknowledged. Findings rely primarily on self-reported data and are therefore subject to recall and social desirability bias. The cross-sectional design and absence of a counterfactual group preclude causal inference. Publication outputs could be verified where ORCID or Google Scholar records were available, but other outcomes were not independently validated. In addition, the predominance of respondents from the WHO African Region reflects the programme's historical orientation and limits generalisability across all LMIC regions. The descriptive analytical approach also did not assess statistical associations between demographic characteristics and outcomes.

Contribution to development practice

This study contributes to development practice by providing empirically grounded insights into how fellowship-based capacity strengthening interacts with institutional environments in resource constrained settings. It highlights human resource leadership, institutional absorptive capacity, and South–South collaboration as critical enablers of sustainable, locally led research systems. These insights are directly relevant to development agencies, funders, and national governments designing long-term research capacity-strengthening interventions.

Conclusion

This study provides descriptive insights into the reported mid- and long-term professional trajectories of WHO/TDR CRDF alumni, particularly within African LMIC contexts. Findings

suggest that many alumni progressed into research leadership roles, sustained scientific productivity, contributed to institutional research capacity, and remained engaged in collaborative research networks. At the same time, persistent structural constraints, including limited funding and institutional support, may affect the sustainability of these gains.

These findings highlight the potential value of pairing individual fellowships with re-entry support, protected research time, stronger research management systems, and South–South collaboration to support more sustainable and locally led research ecosystems.

List of abbreviations

CRDF: Clinical Research and Development Fellowship; **ESSENCE**: Enhancing Support for Strengthening the Effectiveness of National Capacity Efforts; **IQR**: Interquartile Range; **LMICs**: Low- and Middle-Income Countries; **MD**: Doctor of Medicine; **NGO**: Non-Governmental Organization; **ORCID**: Open Researcher and Contributor ID; **PhD/DPhil**: Doctor of Philosophy: **PI**: Principal Investigator; **R**: R Statistical Software; **SDGs**: Sustainable Development Goals; **TDR**; Special Programme for Research and Training in Tropical Diseases; **TPO**: Training Partner Organization; **USD**: United States Dollar; **WHO**: World Health Organization

Declarations

Ethics approval and consent to participate

Participation was voluntary, and respondents were permitted to withdraw at any time. Ethical considerations included informed consent at the start of the survey, where participants were notified of the study's purpose. To ensure confidentiality, all individual data were restricted to the study team, and data was anonymized.

Consent for publication

Not applicable

Availability of data and materials

The data would be availabe anytime up on request.

Competing interests

The authors declared no conflict of interest.

Funding

This study did not receive specific funding. The analysis was conducted as part of monitoring and evaluation of the WHO/TDR CRDF programme. TDR received funding from BMGF for this programme. The funders had no role in the study design, data collection, analysis, interpretation, or writing of the manuscript.

Authors' contributions

MV conceived the study. MV, DGA, PL, NC contributed to study design. MV and DGA contributed to data collection. MV and DGA conducted data cleaning and analysis. MV and DGA drafted the manuscript. NC and PL critically reviewed the manuscript and supervised the conduct of the study. All authors contributed to interpretation of the findings, critically revised the manuscript for intellectual content, and approved the final version.

Acknowledgements

The authors thank the WHO/ TDR for providing access to programme records and for supporting research capacity strengthening in low- and middle-income countries. We also acknowledge all Clinical Research and Development Fellowship alumni for their active participation in the survey.

References

1. Vos T, Lim SS, Abbafati C, Abbas KM, Abbasi M, Abbasifard M, et al. Global burden of 369 diseases and injuries in 204 countries and territories, 1990–2019: a systematic analysis for the Global Burden of Disease Study 2019. The Lancet [Internet]. 2020;396(10258):1204–22. Available from: https://www.sciencedirect.com/science/article/pii/S0140673620309259
2. Abimbola S, Pai M. Will global health survive its decolonisation? The Lancet [Internet]. 2020 Nov 21;396(10263):1627–8. Available from: https://doi.org/10.1016/S0140-6736(20)32417-X
3. WHO. Geneva: World Health Organization. World Health Organization. 2012 [cited 2025 Dec 28]. p. 53 The WHO strategy on research for health. Available from: https://www.afro.who.int/sites/default/files/2020-11/WHO_Strategy_on_research_for_health.pdf
4. AU, AFRICA CDC. Partnerships for African Vaccine Manufacturing (PAVM) Framework for Action | 2022 (Version 1). 2022;2022(Version 1):1–99. Available from: https://africacdc.org/download/partnerships-for-african-vaccine-manufacturing-pavm-framework-for-action/
5. PATH. Market failures and opportunities for increasing access to diagnostics in LMICs. 2022; Available from: https://www.path.org/resources/market-failures-and-opportunities-for-increasing-access-to-diagnostics-in-low-and-middle-income-countries/
6. Pang T, Sadana R, Hanney S, Bhutta ZA, Hyder AA, Simon J. Knowledge for better health: a conceptual framework and foundation for health research systems. Bull World Health Organ. 2003;81:815–20.
7. WHO. Geneva: World Health Organization. Who. 2022 [cited 2025 Dec 29]. Covid-19 Strategic Preparedness. Available from: https://www.who.int/publications/i/item/WHO-WHE-2021.02
8. TDR. Strengthening research capacity [Internet]. [cited 2025 Dec 20]. Available from: https://tdr.who.int/home/our-work/strengthening-research-capacity
9. TDR (Special Programme for Research and Training in Tropical Diseases). Clinical Research Leadership programme [Internet]. [cited 2025 Dec 20]. Available from: https://tdr.who.int/home/our-work/strengthening-research-capacity/clinical-research-and-development-fellowship
10. TDR (Special Programme for Research and Training in Tropical Diseases). TDR Clinical Research & Development Fellowships Call for Application [Internet]. 2012 [cited 2025 Dec 20]. Available from: https://campus.paho.org/en/tdr-clinical-research-development-fellowships-call-application
11. Halpaap B, Vahedi M, Certain E, Alvarado T, Saint Martin C, Merle C, et al. Tracking the career development of scientists in low- and middle-income countries trained through TDR's research capacity strengthening programmes: Learning from monitoring and impact evaluation. PLoS Negl Trop Dis. 2017;11(12):1–15.
12. ESSENCE on Health Research. Essence Good Practice Document Series. 2014 [cited 2025 Dec 28]. p. 1–36 Seven principles for strengthening research capacity in low- and middle-income countries: simple ideas in a complex world. Available from: http://www.who.int/tdr/publications/seven-principles/en/

13. R core Team. R: A Language and Environment for Statistical Computing. R Foundation for Statistical Computing, Vienna, Austria [Internet]. 2024 [cited 2025 Sep 8]. Available from: https://www.r-project.org/.
14. WHO. Geneva: World Health Organization. WHO regional offices [Internet]. [cited 2025 Dec 29]. Available from: https://www.who.int/about/who-we-are/regional-offices
15. Toma M, Blamey A, Mahal D, Gray NM, Allison L, Thakore S, et al. Multi-method evaluation of a national clinical fellowship programme to build leadership capacity for quality improvement. BMJ Open Qual. 2020;9(4):1–10.
16. World Health Organization (WHO). Global strategy on human resources for health: Workforce 2030 [Internet]. Who. 2016. Available from: https://www.who.int/hrh/resources/global_strategy_workforce2030_14_print.pdf?ua=1
17. Beran D, Byass P, Gbakima A, Kahn K, Sankoh O, Tollman S, et al. Research capacity building obligations for global health partners. Lancet Glob Health [Internet]. 2017;5(6):e567–8. Available from: http://dx.doi.org/10.1016/S2214-109X(17)30180-8
18. Kang SSY, Tadesse BT, Jeon HJ, Fallah MP, Dereje N, Ilesanmi OS, et al. Enhancing Clinical Trial Sites in Low- and Middle-Income Countries to Facilitate Product Development in Response to the COVID-19 Pandemic. Clin Infect Dis. 2025 Jul;80(Supplement_1):S1–8.
19. Mahoney R, Hotez PJ, Bottazzi ME. Global regulatory reforms to promote equitable vaccine access in the next pandemic. PLOS global public health. 2023;3(10):e0002482.
20. Nsanzimana S, Rawat A, Wilson LA, Forrest JI, Reis G, Ramagopalan S, et al. Toward a New Paradigm of North-South and South-South Partnerships for Pandemic Preparedness: Lessons Learned from COVID-19 and Other Outbreaks. Am J Trop Med Hyg. 2022 Dec;107(6):1162–5.

List of figures

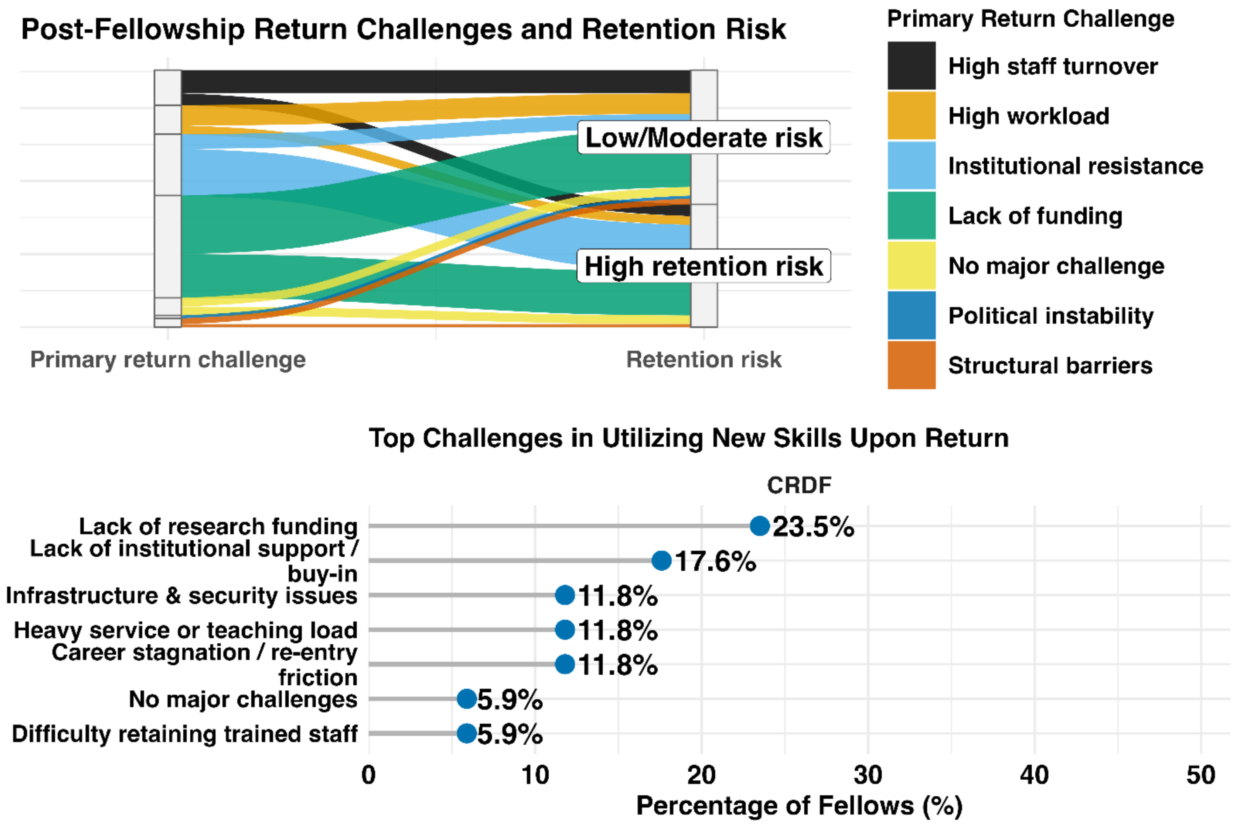

Figure 1: **Post -fellowship return challenges and retention risk and challenges in utilizing new skill upon return**

List of tables

Table 1: Demographic and professional characteristics of fellowship recipients (N=88)

Characteristics	N = 88
**Year of fellowship start**	
2000-2010	9 (10.2%)
2010-2014	19 (21.6%)
2015-2018	31 (35.2%)
2019-2023	29 (32.9%)
**Current citizenship by subregion**	
Eastern Africa	30 (34.1%)
Eastern Asia	1 (1.1%)
Middle Africa	7 (7.9%)
Northern Africa	2 (2.3%)
South America	7 (7.9%)
South-Eastern Asia	1 (1.1%)
Southern Africa	1 (1.1%)
Southern Asia	7 (7.9%)
Western Africa	30 (34.1%)
Western Europe	2 (2.3%)
**Highest academic degree before fellowship**	
Master's	26 (29.5%)
MD	34 (38.6%)
Medical specialist	1 (1.1%)
PhD	27 (30.7%)
**Highest academic degree after fellowship**	
Fellow of the higher education academy (FHEA)	1 (1.1%)
Master's/Diploma	28 (31.8%)
MD	6 (6.8%)
Medical specialist	1 (1.1%)
None	4 (4.5%)
PhD/DPhil	46 (52.2%)
Postdoctoral	1 (1.1%)
**Home institution type before fellowship**	
Hospital/Clinical Setting	8 (9.1%)
Ministry of Health/Government agency	8 (9.1%)
National research institute	24 (27.2%)
NGO/Non-profit	10 (11.4%)
Another research institute	1 (1.1%)
Research institution	1 (1.1%)
University/Academic institution	36 (41.0%)
**Home institution after fellowship**	
Hospital/Clinical Setting	3 (3.4%)
International research institute	6 (6.8%)
Ministry of Health/Government agency	8 (9.1%)
National research institute	22 (25.0%)
NGO/Non-Profit	12 (13.6%)
Pharmaceutical companies	5 (5.7%)
Private research center	-
Product development companies	1 (1.1%)
Other (not specific)	-
Unemployed	1 (1.1%)

University/Academic institution	30 (34.1%)
**Current residence**	
Eastern Africa	30 (34.1%)
Eastern Asia	1 (1.1%)
Middle Africa	7 (8.0%)
Northern Africa	2 (2.3%)
South America	7 (8.0%)
South-Eastern Asia	1 (1.1%)
Southern Africa	1 (1.1%)
Southern Asia	7 (8.0%)
Western Africa	30 (34.1%)
Western Europe	2 (2.3%)

Table 2: Post-fellowship outcomes: Clinical research capacity, competitive funding, and policy engagement (N=88)

Characteristics	N=88
**Extent to which the re-entry grant strengthened clinical research capacity**	
Not at all enabled	16 (18.2%)
Slightly enabled	-
Moderately enabled	18 (20.5%)
Substantially enabled	35 (39.8%)
Fully enabled	19 (21.6%)
**Initiatives implemented at home institution post-fellowship**[1]	
Clinical trial activities	3 (3.4%)
Policy & government advisory	23 (26.1%)
SOPs	61 (69.3%)
Training programs	67 (76.1%)
Other (Not specific)	18 (20.5%)
**Competitive research grants obtained post-fellowship**	
Yes	49 (55.7%)
No	39 (44.3%)
**Number of national grants obtained post-fellowship**	**N=49**
1	13 (26.5%)
2	9 (18.4%)
3	4 (8.2%)
4	1 (2.0%)
5+	4 (8.2%)
Missing	8 (16.3%)
**Number of international grants obtained post-fellowship**	**N=49**
1	17 (34.7%)
2	7 (14.3%)
3	8 (16.3%)
4	2 (4.1%)
5+	9 (18.4%)
Missing	6 (12.2%)
**Amount of grant in USD**<br>**Median (IQR1-IQR3)**<br>**[Min-Max]**	512,320 (111,629-1,200,000)<br>[700-70,000,000]
**Type of grant**[1]	**N=49**
Capacity building grant (for training/infrastructure)	28 (57.1%)
Research grant (for a specific study)	42 (85.7%)
Research grant; Capacity building grant (combined)	18 (36.7%)
**Have you published or produced a policy brief or policy document based on your research findings?**	**N = 88**
Yes	29 (33.0%)
No	59 (67.0%)
**How many policies brief or policy document published or produced**	**N=29**
1	7 (24.1%)
2	14 (48.3%)
4	1 (3.4%)
6	1 (3.4%)
15	1 (3.4%)
Multiple (Number not specified)	5 (17.2%)
**How many implemented**	**N=29**

1	7 (24.1%)
**2**	10 (34.5%)
3	1 (3.4%)
Multiple (Number not specified)	11 (37.9%)

[1]Participants could select multiple answers; percentages are calculated within each fellowship type.

Table 3: Collaboration with the training partner organization after fellowship

**Collaboration with TPO after fellowship**	**N = 88**
Collaboration with individual members of the TPO (e.g., supervisors, mentors, lab teams)	11 (12.5%)
Collaboration with partners or networks you were introduced to through the TPO during the fellowship	4 (4.5%)
Collaboration with partners or networks you were introduced to through the TPO during the fellowship	7 (8.0%)
Direct collaboration with the TPO	4 (4.5%)
Direct collaboration with the TPO; Collaboration with individual members of the TPO (e.g., supervisors, mentors, lab teams)	9 (10.2%)
Direct collaboration with the TPO; Collaboration with individual members of the TPO (e.g., supervisors, mentors, lab teams); Collaboration with partners or networks you were introduced to through the TPO during the fellowship	14 (16.9%)
Direct collaboration with the TPO; Collaboration with partners or networks you were introduced to through the TPO during the fellowship	7 (8.0%)
Direct collaboration with the TPO; The African Field Epidemiology Network (AFENET) and the Institute Pasteur de Dakar of Senegal	1 (1.1%)
Other collaboration; Direct collaboration with the TPO; Was later hired as a Clinical Science Lead in my training institution in Vaccines R&D.	1 (1.1%)
Other collaboration; EDCTP Institutional Capacity Building grant and the Malaria development consortium grant	1 (1.1%)
No collaboration	29 (33.5%)
**Nature of collaboration with TPO[1]**	**N=59**
Co-authored publications	15 (25.4%)
Joint research projects (including clinical trials)	14 (23.7%)
Grant proposal development / submissions	12 (20.3%)
Training activities / capacity building	11 (18.6%)
Mentorship (formal or informal)	9 (15.3%)
Consultancy / expert advisory roles	6 (10.2%)
Employment or continued engagement with TPO	5 (8.5%)
Networking / participation in panels, symposia, programs	4 (6.8%)
**Competitive funding obtained through fellowship networks**	**N = 88**
Yes	27 (30.7%)
No	61 (72.3%)
**Estimate the number of grants/contracts secured through this network**	**N=27**

1	12 (44.4%)
2	7 (25.9%)
3	5 (18.5%)
4	2 (7.4%)
5	1 (3.7%)
No response	-
**Estimate the number of grants/contracts secured through this network in USD**	**N=25**
**Median (IQR1-IQR3)**<br>**[Min-Max]**	300,000 (50,000-850,000)<br>[4,500-100,000,000]
**Type of collaboration (North-North / North-South)**	**N = 88**
Never	8 (9.1%)
Rarely	12 (13.6%)
Sometimes	31 (35.2%)
Very often	37 (42.0%)
**Type of collaboration (South-South)**	
Never	8 (9.1%)
Rarely	17 (19.3%)
Sometimes	33 (37.5%)
Very often	30 (34.1%)
**Type of collaboration (National)**	
Never	4 (4.5%)
Rarely	16 (18.2%)
Sometimes	32 (36.4%)
Very often	36 (40.9%)
**Evolution in authorship**	**N = 88**
**Evolution in first authorship positions after the fellowship compared to before.**	
No change	13 (14.8%)
Minimal improvement	5 (5.7%)
Moderate improvement	12 (13.6%)
Substantial improvement	22 (22.0%)
Significant improvement	36 (40.9%)
**Evolution in last/senior authorship positions after the fellowship compared to before.**	
No change	25 (28.4%)
Minimal improvement	7 (8.0%)
Moderate improvement	13 (14.8%)
Substantial improvement	17 (19.3%)
Significant improvement	26 (29.5%)
**Evolution in corresponding authorship positions after the fellowship compared to before.**	
No change	13 (14.8%)
Minimal improvement	4 (4.5%)
Moderate improvement	13 (14.8%)
Substantial improvement	27 (30.7%)
Significant improvement	31 (35.2%)

Table 4: **Current professional roles and supervisory responsibilities of fellows (N=88)**

**Current position of the fellows[1]**	**N=88**
Academic faculty (Lecturer / Assistant Professor / Professor)	27 (30.7%)
Associate Director General	1 (1.1%)
Associate Director of NGO	1 (1.1%)
Biostatistician	1 (1.1%)
Chairperson of a Research Ethics Committee	1 (1.1%)
Clinical Data Manager	1 (1.1%)
Clinical Development Physician (Director level in the Pharmaceutical Industry)	1 (1.1%)
Clinical Research Scientist/ Clinical Science Lead/Safety Physician	1 (1.1%)
Clinical Trial Coordinator	11 (12.5%)
Clinical Trial Manager	8 (9.1%)
Clinical trial data unit team lead	1 (1.1%)
Disease Surveillance Information System Coordinator	1 (1.1%)
Expert and Lead	1 (1.1%)
Head of Department (university, research institute, or hospital)	16 (18.2%)
Head of Research Unit	14 (15.9%)
Head of clinical department	1 (1.1%)
Lead for a Clinical Research	1 (1.1%)
Market Development in clinical research	1 (1.1%)
Policy Advisor/Maker/Implementer	10 (11.4%)
Post-doctoral Researcher	1 (1.1%)
Principal Investigator (PI) / Lead Researcher	38 (43.2%)
Program Coordinator of the Epidemiology and Disease Control Program, heading the PHEOC and also the Program Director for The Gambia FETP Program	1 (1.1%)
Project Manager (research or public health project)	18 (20.4%)
Research Director	10 (11.4%)
Research Group Leader / Team Leader	28 (31.8%)
Responsible for clinical operations	1 (1.1%)
Safety lead for a global vaccine program	1 (1.1%)
Strategy and business development in clinical research	1 (1.1%)
Technical Officer in Foresight Leadership and Ethics in Science Unit	1 (1.1%)
Therapeutic area leads drug safety (Exec Director) in a pharmaceutical company	1 (1.1%)
**Direct management of other research staff/students**	
Yes	70 (79.5%)
No	18 (20.5%)
**Number of staff/students managing by the fellow**	**N=70**
1	1 (1.4%)
2-5	23 (32.9%)
6-10	17 (24.3%)
11-20	12 (17.1%)
21-50	4 (5.7%)
51	4 (5.7%)
No response	18 (25.7%)
**Level of staff/students managed by the fellow**	**N=70**
Junior/Early career	1 (1.1%)

Mid-level/MSc	4 (5.7%)
PhD/MD level	4 (5.7%)
Unspecified level	25 (35.7%)
No response	36 (51.4%)
**Direct supervision of other research staff/students**	
Yes	70 (79.5%)
No	18 (20.5%)
**Number of s staff/students supervised by the fellow**	**N=70**
1–2	7 (10.0%)
3–5	22 (31.4%)
6–10	20 (28.6%)
11–20	10 (14.3%)
20+	2 (2.9%)
No response	9 (12.9%)
**Level of staff/students supervised by the fellow**	**N=70**
Junior/Early career	22 (31.4%)
Mid-level/MSc	9 (12.9%)
PhD/MD level	5 (7.1%)
Unspecified level	5 (7.1%)
No response	29 (41.4%)

[1]Participants could select multiple roles; percentages are calculated within each fellowship type.

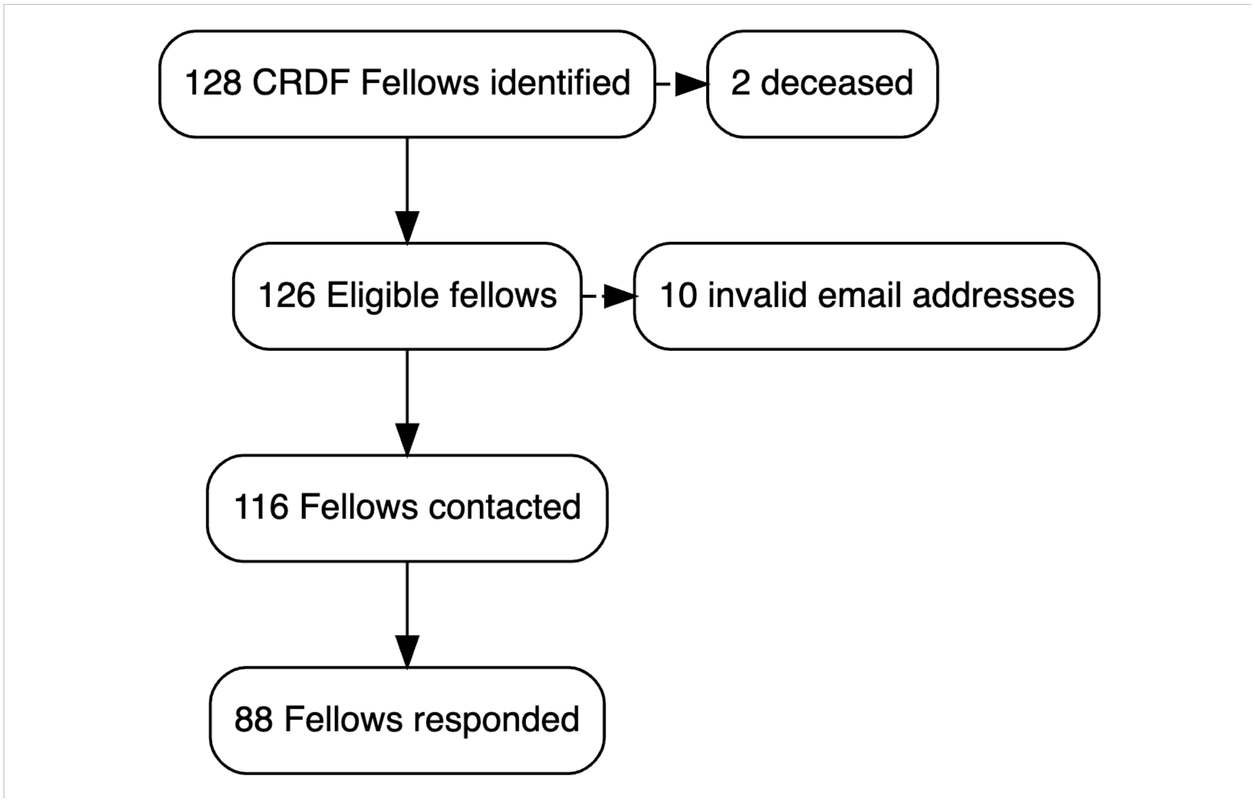

Supplemantry figure 1: Flow diagram.

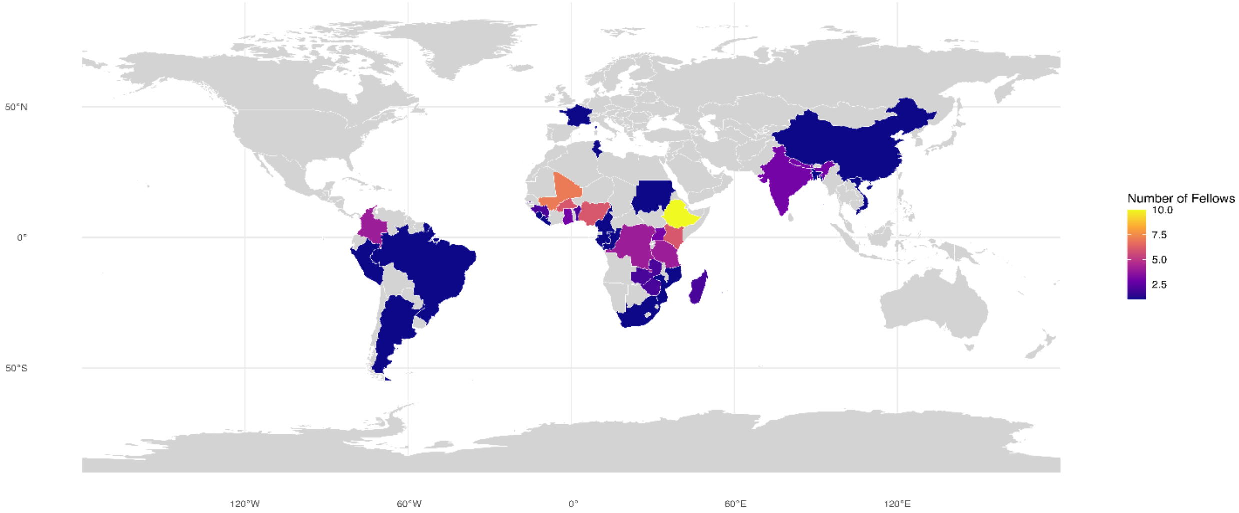

Supplemantry figure 2: Citizenship of CRDF fellows.

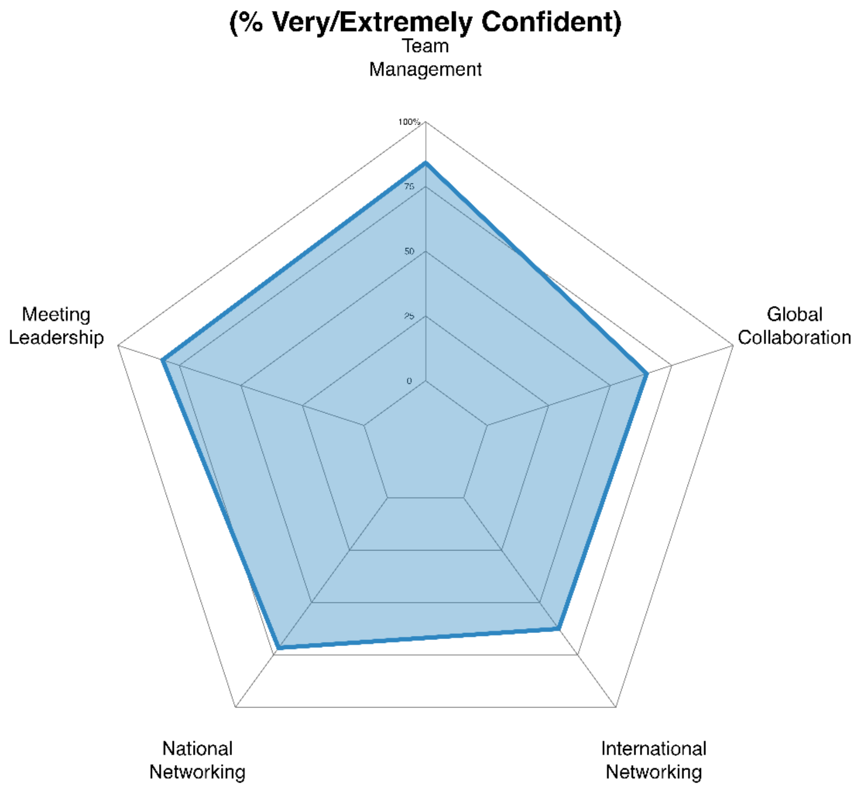

Supplementary figure 3: Confidence in leadership skills during and after fellowship.

Supplementary table 1: Leadership confidence in applying clinical research leadership skills acquired during the fellowship (N=88)

Characteristics	N = 88
**TDR skills used in current job**	
Yes	87 (98.9%)
No	-
Missing	1 (1.1%)
**Strategic planning for clinical research programs)**	
Not at all confident	-
Slightly confident	3 (3.4%)
Moderately confident	19 (21.5%)
Very confident	38 (43.2%)
Extremely confident	28 (31.8%)
**Mentoring and coaching junior researchers/staff**	
Not at all confident	-
Slightly confident	1 (1.1%)
Moderately confident	18 (20.5%)
Very confident	25 (28.4%)
Extremely confident	44 (50.0%)
**Supervising junior researchers/staff**	
Not at all confident	-
Slightly confident	-
Moderately confident	14 (15.9%)
Very confident	25 (28.4%)
Extremely confident	49 (55.7%)
**Effective stakeholder engagement (e.g., policymakers, regulators)**	
Not at all confident	2 (2.3%)
Slightly confident	3 (3.4%)
Moderately confident	25 (28.4%)
Very confident	32 (36.4%)
Extremely confident	26 (29.5%)
**Managing Budgets and financial governance for programs**	
Not at all confident	2 (2.3%)
Slightly confident	3 (3.4%)
Moderately confident	25 (28.4%)
Very confident	32 (36.4%)
Extremely confident	26 (29.5%)
**Leading a multidisciplinary clinical trial team**	
Not at all confident	1 (1.1%)
Slightly confident	7 (7.9%)
Moderately confident	19 (21.6%)
Very confident	26 (29.5%)
Extremely confident	35 (39.8%)
**Developing a clinical trial protocol**	
Not at all confident	1 (1.1%)
Slightly confident	4 (4.5%)
Moderately confident	18 (20.5%)
Very confident	31 (35.2%)
Extremely confident	34 (38.6%)

Supplementary table 2: Confidence in leadership skills during and after fellowship (N=88)

**Leadership skill**	N = 88
**Managing a team**	
Slightly confident	1 (1.1%)
Moderately confident	13 (14.7%)
Very confident	35 (39.8%)
Extremely confident	39 (44.3%)
**Leading meetings effectively**	
Slightly confident	4 (4.5%)
Moderately confident	12 (13.6%)
Very confident	30 (34.1%)
Extremely confident	42 (47.7%)
**Networking with national stakeholders (e.g., ministries, funders)**	
Slightly confident	3 (3.4%)
Moderately confident	22 (25.0%)
Very confident	38 (43.2%)
Extremely confident	25 (28.4%)
**Networking with international stakeholders (e.g., WHO, global partners)**	
Slightly confident	7 (8.0%)
Moderately confident	26 (29.5%)
Very confident	30 (34.1%)
Extremely confident	25 (28.4%)
**Coordinating multi-disciplinary or multi-country collaborations**	
Not at all confident	1 (1.1%)
Slightly confident	9 (10.2%)
Moderately confident	21 (23.9%)
Very confident	29 (33.0%)
Extremely confident	28 (31.8%)

Supplementary table 3: Importance of mentorship and career support for professional goals (N=88)

**Quality mentorship**	**N=88**
**Quality mentorship received during fellowship**	
Poor	2 (2.3%)
Fair	3 (3.4%)
Good	11 (12.5%)
Very good	29 (33.0%)
Excellent	43 (48.8%)
**Usefulness of post-fellowship mentorship**	
Not useful	3 (3.4%)
Slightly useful	4 (4.5%)
Moderately useful	8 (9.1%)
Very useful	34 (38.6%)
Essential	39 (44.3%)
**Overall career support (Importance)**	
**TDR / The fellowship program**	
Not at all	1 (1.1%)
Slightly important	1 (1.1%)
Moderately important	5 (5.7%)
Important	4 (4.5%)
Very important	25 (28.4%)
Extremely important	52 (59.1%)
**Supervisor (during fellowship)**	
Not at all	6 (6.8%)
Slightly important	7 (8.0%)
Moderately important	6 (6.8%)
Important	1 (1.1%)
Very important	30 (34.1%)
Extremely important	38 (43.2%)
**Employer/Home institution**	
Not at all	4 (4.5%)
Slightly important	5 (5.7%)
Moderately important	19 (21.6%)
Important	3 (3.4%)
Very important	27 (30.7%)
Extremely important	30 (34.1%)
**Other funding agencies (non-TDR)**	
Not at all	16 (18.2%)
Slightly important	10 (11.4%)
Moderately important	16 (18.2%)
Important	10 (11.4%)
Very important	22 (25.0%)
Extremely important	14 (15.9%)
**Support from mentor (During fellowship]**	
Not at all	6 (6.8%)
Slightly important	3 (3.4%)
Moderately important	10 (11.4%)
Important	4 (4.5%)
Very important	25 (28.4%)
Extremely important	40 (45.5%)
**Support from peers/professional network**	

Not at all	1 (1.1%)
Slightly important	15 (17.0%)
Moderately important	20 (22.7%)
Important	2 (2.2%)
Very important	25 (28.4%)
Extremely important	25 (28.4%)
**Family/Personal support system**	
Not at all	-
Slightly important	3 (3.4%)
Moderately important	12 (13.6%)
Important	3 (3.4%)
Very important	19 (21.6%)
Extremely important	51 (58.0%)

Supplementary table 4: Equity, support, and barriers per-and post-fellowship (N=88)

**Pre-fellowship financial/institutional barriers**	**N = 88**
Yes	24 (27.3%)
No	64 (72.7%)
**Pre-fellowship financial/institutional barrier type**	**N=24**
Home Institution release/commitment	12 (50.0%)
Government/Ministry approval	4 (16.7%)
Lack of initial funding for family/relocation	8 (33.3%)
**Challenges during fellowship placement**[1]	**N = 88**
Housing & banking (accommodation, bank accounts)	29 (33.0%)
Visa & travel (applications, family visas, documents)	23 (26.1%)
COVID-19 related (lockdowns, seclusion)	5 (5.7%)
Institutional/TPO support (guidance, discrimination, mentorship)	3 (3.4%)
Language & integration (language barriers, cultural fit)	1 (1.1%)
Academic/ miscellaneous (Student registration, mid-term breaks, RAS)	1 (1.1%)
No challenges	14 (15.9%)
Missing	12 (13.6%)
**Resource equity at TPO**	
Yes, fully equitable	47 (53.4%)
Mostly equitable	29 (33.0%)
Somewhat inequitable	8 (9.1%)
Very inequitable	1 (1.1%)
Other: Covid-19 posed a challenge	1 (1.1%)
Other: Establishment joined after program was well resourced	1 (1.1%)
Missing	1 (1.1%)
**Institutional support for re-entry projects**	
Yes, fully supported	40 (45.5%)
Somewhat supported	26 (29.5%)
No, faced significant resistance	9 (10.2%)
Not Applicable	13 (14.8%)
**Retention risk: intent to move abroad**	
Minimal risk - not at all	18 (20.5%)
Low risk	9 (10.2%)
Moderate risk / undecided	19 (21.6%)
Significant risk	21 (23.9%)
High risk - actively pursuing opportunities abroad	21 (23.9%)

Top challenges in utilizing new skills upon return[2]	
Lack of research funding	35 (39.8%)
Lack of institutional support / buy-in	18 (20.5%)
Heavy service or teaching load	10 (11.4%)
Difficulty retaining trained staff	12 (13.6%)
Career stagnation / re-entry friction	3 (3.4%)
Infrastructure & security issues	-
No major challenges	11 (12.5%)
Missing	1 (1.1%)
**Any personal/structural challenges related to gender, family responsibilities, or institutional culture that impacted the ability to fully leverage the fellowship opportunity**	
Yes	19 (21.6%)
No	69 (78.4%)
**What specific support mechanism could TDR/the TPO have provided to better mitigate this challenge?**	
Family & relocation support (Visas for spouse, family travel, maternity care)	10 (11.4%)
Reintegration & career support (funding bridge, institutional advocacy, placement)	6 (6.8%)
Institutional culture & equity (anti-discrimination, professional status vs student)	2 (2.2%)
Psychological & social support (counseling, cultural orientation, loneliness)	1 (1.1%)
No challenges reported	69 (78.4%)

[1]Total reflects that some respondents selected multiple overlapping categories.
[2]Total slightly higher for CDRF as one respondent's "Other" text spanned multiple themes.

Supplementary table 5: Critical skills not sufficiently covered during the fellowship but essential for short-term reintegration (N = 88)

Theme	Sub-theme	Responses	Response counts (N= 88)
**Operations, site management, and logistics**	Site/Trial management	Site and trial operations; Study and site management; Protocol operationalization; Review protocols and conduct feasibility planning, risk assessments; Participation in hands-on clinical trials; Site-level study management (logistics, staff, local regulatory/ethical processes)	8 (9.1%)
	Research operations (General)	Research operations; Protocol operationalization; Protocol development	4 (4.5%)
	Laboratory work	Hands-on experience of performing laboratory assays; Laboratories technician (due to lack of time)	3 (3.4%)
**Leadership, governance, and project/Program management**	Leadership/Management	Advanced leadership and programme management (strategic oversight, multisectoral coordination, decision-making); Strategic leadership; Leadership and Governance; Management of conflict; Mentoring and coaching juniors' scientists	7 (7.9%)
	Project management	Project management; Project Management; Research project management and evaluation	4 (4.5%)
	Soft skills/Transversal	Soft skills; Communication; Adaptability/Organizational change; Maintaining a network	4 (4.5%)

**Financial and resource management (Grants)**	Grant acquisition	Grant application; Grant writing; Grants; Grants writing skills; Obtaining funding; Development of a competitive clinical research grant proposal; Securing funding and engaging with sponsors	11 (12.5%)
	Financial management	Budget and financial planning; Financial management; Financial planning; Resource Management; Resources management (human and financial); Financial Governance for programs; Planning a clinic trial study financially	10 (11.4%)
**Data management and biostatistics**	Data/Biostatistics	Data analysis and interpretation; Data management and analysis; Data management skills; Design and planning of research; Interpretation of study results; Statistics	6 (6.8%)
**Regulatory, ethics, and stakeholder communication**	Regulatory/Governance	Regulation and Governance; Regulations and governance; Interaction with regulators; Stakeholder and regulatory communication skills (ethics committees, national authorities); Intellectual Property rights and Patenting	6 (6.8%)
	Stakeholder/Public engagement	Interaction with public & study participants; Communication; Communicating with policy makers; Community engagement; Training and discussion with healthcare workers and public speaking	6 (6.8%)
**Specialized research and trial design**	Specialized skills	Adaptive designs in RCTs; Vaccine development/research; Phase 1 clinical trial experience; Pharmacovigilance/Post-marketing surveillance; Integration of genomics and bioinformatics	6 (6.8%)
**Other**	No gaps/Fully covered	All essential skills properly covered; All skills were covered during fellowship; I can't think of skills that were not adequately covered; I see none; was sufficient for short term reintegration plan; The foundation I got was built during my fellowship.	7 (7.9%)
	Other responses	N/A; None; Nothing comes to mind; Not applicable (took a different path); Not yet acquired (funding/sponsors); Returned to position no longer available.	23 (26.1%)

Supplementary table 6: Critical skills not sufficiently covered during the fellowship but essential for long-term reintegration (N = 88)

Theme	Sub-theme	Illustrative responses	Response counts (N= 88)
**Leadership and programme management**	Strategic leadership and career development	Clinical research leadership; leading research groups; long-term career strategy	7 (8.0%)
	Project and programme management	Programme coordination; strategic project leadership	5 (5.7%)
	Team and people management	Conflict resolution; supervision and mentorship; staff management; virtual leadership	8 (9.1%)

**Financial strategy and resource mobilization**	Grant acquisition and writing	Grant opportunities; proposal development; sustaining funding	13 (14.8%)
	Financial and resource management	Budgeting; financial governance; staff and resource management	8 (9.1%)
**Advanced research methods and data**	Data analysis and statistics	Biostatistics; data interpretation; advanced analytics and data science	9 (10.2%)
	Specialized methods	Molecular biology; bioinformatics; pharmacovigilance; product development	6 (6.8%)
**Regulatory and product development**	Product and policy translation	Translating trial findings into policy and practice; post-marketing surveillance	4 (4.5%)
	Regulatory and governance	Ethics, quality, risk management; liability and insurance	4 (4.5%)
**Operational and study management**	Study operations	Protocol operationalization; trial platform setup; monitoring	5 (5.7%)
**Other / transversal skills**	Transversal skills	English language proficiency; networking; cognitive and adaptive skills	3 (3.4%)
	No gaps / not applicable	Skills fully covered; no additional needs identified	33 (37.5%)

Supplementary table 7: Biggest barriers fellows currently face when trying to secure competitive, international research and national grants (PI/Co-PI)

Theme	Sub-theme	Barriers	Response counts (N= 88)
**Grant/Funder knowledge & proposal quality**	Grant opportunity identification	Difficulty identifying suitable funding calls or research partners	62 (70.5%)
	Funder knowledge	Limited knowledge of funder-specific requirements (e.g., EDCTP, NIH, Wellcome, etc.)	
	Proposal preparation skill	Lack of dedicated grant writing training	
	Funder knowledge	Complex application processes	
	Grant opportunity identification	Networking with others research institutes	
**Institutional & administrative support**	Administrative support	Lack of administrative support	36 (40.9%)
	Institutional management support	Limited institutional support for grant management (e.g., finance, contracts, overhead)	
	Institutional resource (Equipment)	Lack of equipment to carry out the planned laboratory assays	
**Competitiveness & track record detailed Barrier phrase (or key component)**	Competitiveness & experience	Limited track record or competitiveness compared to Global North institutions	33 (37.5%)
	Partnership/Equity	Establishing equitable partnership with institutions in global north.	

	Partnership/Networking	Limited partnerships with established consortia	
**Time & workload**	Workload & capacity	Time constraints due to heavy service/teaching load	26 (29.5%)
**Other/Unique barriers**	External/Political	Political barriers due to the actual political situation in my home country	9 (10.2%)
	Career/Policy	Lack of position at the national government	
	Research area specific	Interested in surveillance and using routine and these do not attack much funding.	
**Research capacity & environment**	PI capacity & workload	Time constraints due to heavy service/teaching load; Lack of dedicated grant writing training; PI competitiveness.	45 (51.3%)
	Institutional & administrative support	Administrative support (General); Limited institutional support for grant management (finance, contracts, overhead); Political barriers.	44 (50.0%)
**National funding landscape**	Access & scarcity	Difficulty in identifying appropriate funding calls/partners; Lack of national grant opportunities/scarcity.	38 (43.2%)
	Scope & fit	Too small amount of funding; Grant size/value; Research focus priority/fit.	2 (2.3%)
**Other / Not applicable**	Exclusion/Unspecified	Not applicable	13 (14.8%)
